# Human CD141^+^ Dendritic Cell and CD1c^+^ Dendritic Cell Undergo Concordant Early Genetic Programming after Activation in Humanized Mice *In Vivo*

**DOI:** 10.3389/fimmu.2017.01419

**Published:** 2017-10-30

**Authors:** Yoshihito Minoda, Isaac Virshup, Ingrid Leal Rojas, Oscar Haigh, Yide Wong, John J. Miles, Christine A. Wells, Kristen J. Radford

**Affiliations:** ^1^Cancer Immunotherapies Laboratory, Mater Research Institute, University of Queensland, Translational Research Institute, Brisbane, QLD, Australia; ^2^The Centre for Stem Cell Systems, Anatomy and Neuroscience, Faculty of Medicine, Dentistry and Health Sciences, The University of Melbourne, Melbourne, VIC, Australia; ^3^Centre for Biodiscovery and Molecular Development of Therapeutics, AITHM, James Cook University, Cairns, QLD, Australia; ^4^Walter and Eliza Hall Institute of Medical Research, Melbourne, VIC, Australia

**Keywords:** humanized mice, dendritic cells, CD141^+^ DC, CD1c^+^ DC, poly I:C, R848, microarray

## Abstract

Human immune cell subsets develop in immunodeficient mice following reconstitution with human CD34^+^ hematopoietic stem cells. These “humanized” mice are useful models to study human immunology and human-tropic infections, autoimmunity, and cancer. However, some human immune cell subsets are unable to fully develop or acquire full functional capacity due to a lack of cross-reactivity of many growth factors and cytokines between species. Conventional dendritic cells (cDCs) in mice are categorized into cDC1, which mediate T helper (Th)1 and CD8^+^ T cell responses, and cDC2, which mediate Th2 and Th17 responses. The likely human equivalents are CD141^+^ DC and CD1c^+^ DC subsets for mouse cDC1 and cDC2, respectively, but the extent of any interspecies differences is poorly characterized. Here, we exploit the fact that human CD141^+^ DC and CD1c^+^ DC develop in humanized mice, to further explore their equivalency *in vivo*. Global transcriptome analysis of CD141^+^ DC and CD1c^+^ DC isolated from humanized mice demonstrated that they closely resemble those in human blood. Activation of DC subsets *in vivo*, with the TLR3 ligand poly I:C, and the TLR7/8 ligand R848 revealed that a core panel of genes consistent with DC maturation status were upregulated by both subsets. R848 specifically upregulated genes associated with Th17 responses by CD1c^+^ DC, while poly I:C upregulated IFN-λ genes specifically by CD141^+^ DC. *MYCL* expression, known to be essential for CD8^+^ T cell priming by mouse DC, was specifically induced in CD141^+^ DC after activation. Concomitantly, CD141^+^ DC were superior to CD1c^+^ DC in their ability to prime naïve antigen-specific CD8^+^ T cells. Thus, CD141^+^ DC and CD1c^+^ DC share a similar activation profiles *in vivo* but also have induce unique signatures that support specialized roles in CD8^+^ T cell priming and Th17 responses, respectively. In combination, these data demonstrate that humanized mice provide an attractive and tractable model to study human DC *in vitro* and *in vivo*.

## Introduction

Dendritic cells (DCs) are specialized antigen (Ag)-presenting cells that initiate and direct immune responses (1, 2). DC are a heterogenous cell population comprised of distinct subsets that harbor specialized capacity to drive specific immune responses. DC develop from CD34^+^ hematopoietic stem cells (HSC) in the bone marrow (BM) that develop into the common myeloid progenitor, giving rise to the more restricted macrophage/DC progenitor (MDP). The MDP can develop into monocytes, or the common DC progenitor (CDP). The CDP give rise to plasmacytoid (p) DC and two “classical” or “conventional” (c) DC subsets now referred to as “cDC1” and “cDC2.” While surface marker expression has been the convention for characterization of conventional DC subsets, gene expression studies are redefining functional characteristics within these subsets (1, 3–5). pDCs are major producers of type-I IFN and are considered important for anti-viral immunity, the cDC1 and 2 subsets are critical for shaping adaptive immunity to intracellular and extracellular pathogens, respectively.

In mice, the cDC1 subset comprises the lymphoid resident CD8^+^ DC and the tissue resident CD103^+^ DC. These DCs require FLT3L and transcription factors IRF8, ID2, and Batf3 for their development. cDC1 are required for priming protective CD8^+^ T cell responses against cancer and for the efficacy of cancer immunotherapies (6). cDC1 produce high levels of IL-12p70, induce T helper (Th)-1 responses, and cross-present exogenous Ag for priming of CD8^+^ cytolytic T cell responses (7–9). Hallmarks of these functions include the expression of chemokine receptor, XCR1, and adhesion molecule nectin-like protein 2 (Necl2/CADM1), both of which play important roles in CD8^+^ T cell priming (10–12). The presence of C-type lectin-like receptor, Clec9A, is essential for recognition and cross-priming of necrotic cell Ag, as is expression of TLR3, which enhances cross-priming and induces large amounts of IFNλ following ligation with agonists such as poly I:C (11, 13–15); and reviewed in Ref. (16). The human equivalent of cDC1 are defined as CD141^+^ (BDCA3)^+^ DC and share many similarities with mouse cDC1, including expression of XCR1, CADM1, Clec9A, and TLR3, Type III IFN production in response to TLR3 ligation, and cross-presentation of Ag from necrotic cells (2). There is therefore a strong rationale to develop similar strategies to target the human cDC1 equivalent for new cancer immunotherapies (16).

The cDC2 subset is also referred to as CD11b^+^ DC in the mouse lymphoid and non-lymphoid tissues (1, 4). These DCs are FLT3L and IRF4 dependent and share significant overlap in phenotype with cells of the monocyte lineage (17). Monocyte-derived DCs contribute to numerous DC niches throughout the body under inflamed conditions [reviewed in Ref. (18)], and are phenotypically distinct from cDC1 and cDC2 by a lack of expression of Flt3, and the zinc finger transcription factor zbtb46 (19). cDC2 play a key role in driving adaptive immune responses to extracellular pathogens, owing to their capacity to promote Th2 and Th17 responses. The human equivalents of mouse cDC2 are defined as CD1c (BDCA-1)^+^ DC (2), and while there is evidence to suggest that they promote Th17 responses (20), they also produce high levels of IL-12, induce Th1 responses and can cross-present some forms of Ag to CD8^+^ T cells (21); in the absence of inducing T regulatory cells (22). Indeed CD1c^+^ DCs have also been trialed as vaccine candidates (23). Thus, there is currently no clear consensus as to whether targeting specific or multiple human DC subsets will be most beneficial for targeted immunotherapy (22).

Effective preclinical models to study human CD141^+^ DC and CD1c^+^ DC *in vivo* are needed in order to further understand fundamental human DC biology and evaluate new immunotherapeutics. Transfer of human CD34^+^ HSC into immunodeficient mice lacking T, B, and NK cells leads to stable long-term engraftment of human HSC and differentiation of human immune cell subsets. These “humanized”(hu) mice are emerging as a powerful tool to study the human immune system and are being increasingly used to model human-tropic infectious diseases, hematopoiesis, autoimmunity, and cancer and to evaluate new drugs, vaccines, and immunotherapeutics (24–26).

One of the current limitations of hu mouse models is the defective development and/or function of some human leukocyte compartments, arising from a lack of cross-reactivity between mouse and human cytokines and growth factors (24–27). This is most notable within the monocyte/macrophage lineages, which require the addition of human cytokines to promote development and acquire functional capacity. Mouse Rag2^−/−^Il2rg^−/−^ strains with human cytokine genes “knocked in” are under development, these strains accommodate enhanced monocyte/macrophage and NK cell lineage development (26). In contrast, we and others have shown that human CD141^+^ and CD1c^+^ DC subsets develop in the BM, spleen, and lungs following human CD34^+^ reconstitution in a number of immunodeficient mouse strains, making this an attractive model to study human cDC function *in vivo* (28–30). Although the CD141^+^ DC and CD1c^+^ DC that develop in these mice exhibit many of the phenotypic and functional characteristics of their human blood counterparts, the extent to which they recapitulate human DC functionally has not been fully defined. In this study, we examined the global transcriptome of the CD141^+^ DC and CD1c^+^ DC that develop and become activated *in vivo* in hu mice to establish the extent of their similarity with their human blood counterparts. We then used this model to identify early changes in gene expression associated with activation of human CD141^+^ DC and CD1c^+^ DC *in vivo*. These data validate hu mice as a powerful model to study human DC and identify common and unique pathways associated with *in vivo* activation.

## Materials and Methods

### Generation of Hu Mice and Isolation of DC

Cord blood was obtained with written informed consent from the Queensland Cord Blood Bank with approval from the Mater Adult Hospital Human Ethics Committee. CD34^+^ hematopoietic progenitor cells were isolated by density gradient enrichment followed by a positive selection using a CD34^+^ isolation kit (Miltenyi Biotec) as previously described (30). NSG-A2 mice (stock no. 014570) were purchased from Jackson Laboratories. 2–5-day-old NSG-A2 pups received 10 Gy total body irradiation 4 h prior to intrahepatic injection of human CD34^+^ cells. Engraftment of human CD45^+^ cells was confirmed 10–12 weeks later, after which hu mice received 2 s.c. doses of human recombinant huFLT3-L (BioXcell) 4 days apart prior to experimentation. Engrafted mice were injected retro-orbitally with 50 µg poly IC (Invivogen) or 20 µg R848 (Invivogen) alone or in combination and mice were euthanized 2 h later. This study was carried out in accordance with the recommendations of the Australian code for the care and use of animals for scientific purposes (8th Edition). The protocol was approved by the University of Queensland Animal Ethics Committee.

### Flow Cytometry

Single cell suspensions of BM, liver, lung, spleen, and peripheral blood from engrafted mice were blocked with rat and mouse serum then labeled with Live Dead^®^ Aqua (Life Technologies), anti-mouse CD45 PerCP Cy5.5, anti-human CD45 APC Cy7, HLA DR PE Cy7, CD19/20 Pacific blue and either CD141 APC, CD123 PE (all from BioLegend), and CD1c FITC (Abcam) to identify DC, or CD3 Pacific blue, CD8 PE Cy7, CD14 APC (all from BioLegend), and CD4 FITC (BD Biosciences) for other leukocytes (Figure S1 in Supplementary Material). Absolute cell counts were determined by the addition of 5,000 Trucount beads (BD Biosciences) per tube. Data were acquired on a Cyan flow cytometer (Beckman Coulter) and analyzed using Flow Jo software (Tree star, version 8).

### DC Isolation from Hu Mice

Human DCs were enriched from single cell BM suspensions by first labeling with Ab specific for human CD3, CD14, CD19, CD20 (all from Beckman Coulter), CD34 (BD BioSciences), and mouse CD45 (BD BioSciences) and Ter119 (BioLegend) followed by depletion of bound cells using sheep anti-rat IgG Dynabeads (Invitrogen) as previously published (30). Cells were then labeled with Live Dead^®^ aqua, anti-mouse CD45 PerCP Cy5.5, anti-human CD45 APC Cy7, CD3/CD14/CD19/CD20 Pacific blue, HLA DR PE Cy7, CD123 PE or PerCP Cy5.5, CD1c FITC, and CD141 APC and sorted using a Moflo Astrios (Beckman Coulter) (Figure S2 in Supplementary Material).

### Gene Expression Analysis

Total RNA from purified DC subsets was prepared by resuspending between 1 × 10^5^ and 1 × 10^6^ cells in TRIzol Reagent (Life Technologies) followed by chloroform extraction and isopropanol precipitation. RNA quantity and integrity was measured using the Bioanalyzer RNA Nano chip (Agilent Technologies), with all RNA integrity numbers ranging between 8.4 and 9.7. cDNA was generated from 450 ng RNA and converted to cRNA using an Illumina TotalPrep RNA Amplification Kit (Ambion). A 14 h *in vitro* transcription was performed. Amplified cRNA (750 ng) for each sample was hybridized onto HumanHT-12 v4 Expression BeadChip Kit (Illumina) according to the manufacturer’s protocol and scanned on an Illumina BeadArray Reader (Illumina). Quality control, normalization, and log 2 transformation of the raw expression data were performed using the *Lumi* Bioconductor package (31) and integrated into the Stemformatics platform www.stemformatics.org (32) for visualization. Transcripts differentially expressed by CD141^+^ DC and CD1c^+^ DC were identified using ANOVA analysis with *p* value cut-off threshold of Bonferroni correction *p* < 0.01 and log(2) 3-fold difference. The data have been deposited in the GEO database, accession # GSE99666, and on Stemformatics http://www.stemformatics.org/datasets/search?ds_id=6612. Publically available data sets and gene signatures for human DC subsets GSE35457 (17) were processed similarly for comparison.

### Cytokine Chemokine and Activation Marker Assays

Dendritic cell subsets purified from hu mice were exposed to TLR adjuvants for 18 h *in vitro*, or cultured in media alone for 18 h. Costimulatory molecule expression on the cell surface was quantified by flow cytometry. Culture supernatants and serum samples from the mice were analyzed for human cytokines using a LEGENDplex™ flow cytometry bead array kit (Biolegend). Human IFN-λ was measured by ELISA (R&D Systems).

### CD8^+^ T Cell Priming Assays

HLA-A2^+^ DC subsets isolated from hu mice were pulsed with the HLA-A2-restricted MART-1 peptide, ELAGIGILTV (1 µM) in the presence of poly IC and R848 for 2 h at 37°C. Naïve CD8^+^ T cells from autologous cord blood mononuclear cells were purified by negative selection using CD8^+^ T cell isolation kit (Miltenyi Biotec). Peptide-pulsed DCs were cultured with naïve CD8^+^ T cells in the presence of IL-2 (Hoffman-LaRoche) and T cell growth factor for 21 days. MART-1-specific CD8^+^ T cells were quantitated by flow cytometry using a MART-1/HLA-A2-specific pentamer-APC (ProImmune), Live Dead^®^ aqua, and anti-human CD19-Pacific blue, CD14-Pacific blue, CD3-PE, and CD8-FITC antibodies (BioLegend). Samples were acquired on a LSR Fortessa flow cytometer (BD Biosciences) and analyzed using Flow Jo software (Tree star, version 8). For polyfunctional T cell assays, the CD8^+^ T cell cultures were restimulated for 6 h in the presence of absence of MART-1 peptide (1 µM) with brefeldin A, GolgiStop, and anti-CD107a-FITC (BD Biosciences). Cells were then washed and labeled with anti-CD4 BV711, anti-CD8 APC Cy7, and Live dead^®^ followed by permeabilization (Cytofix/Cytoperm; BD Biosciences) and stained with anti-TNFα APC, anti-IFNγ PE, and anti-IL-2 Alexa700. Samples were acquired on a LSR Fortessa cytometer (BD Biosciences) and analyzed using Flow Jo software (Tree star, version 8). Boolean gating was performed to measure the frequency of each response based on all possible functional combinations of IFNγ, TNFα, IL-2, and CD107a. The data were analyzed and graphed using Simplified Presentation of Incredibly Complex Evaluations (SPICE) software, version 5.3.

## Results

### Hu Mouse CD141^+^ DC and CD1c^+^ DC Are Closely Related to Human Blood Equivalents

**Figure 1:**
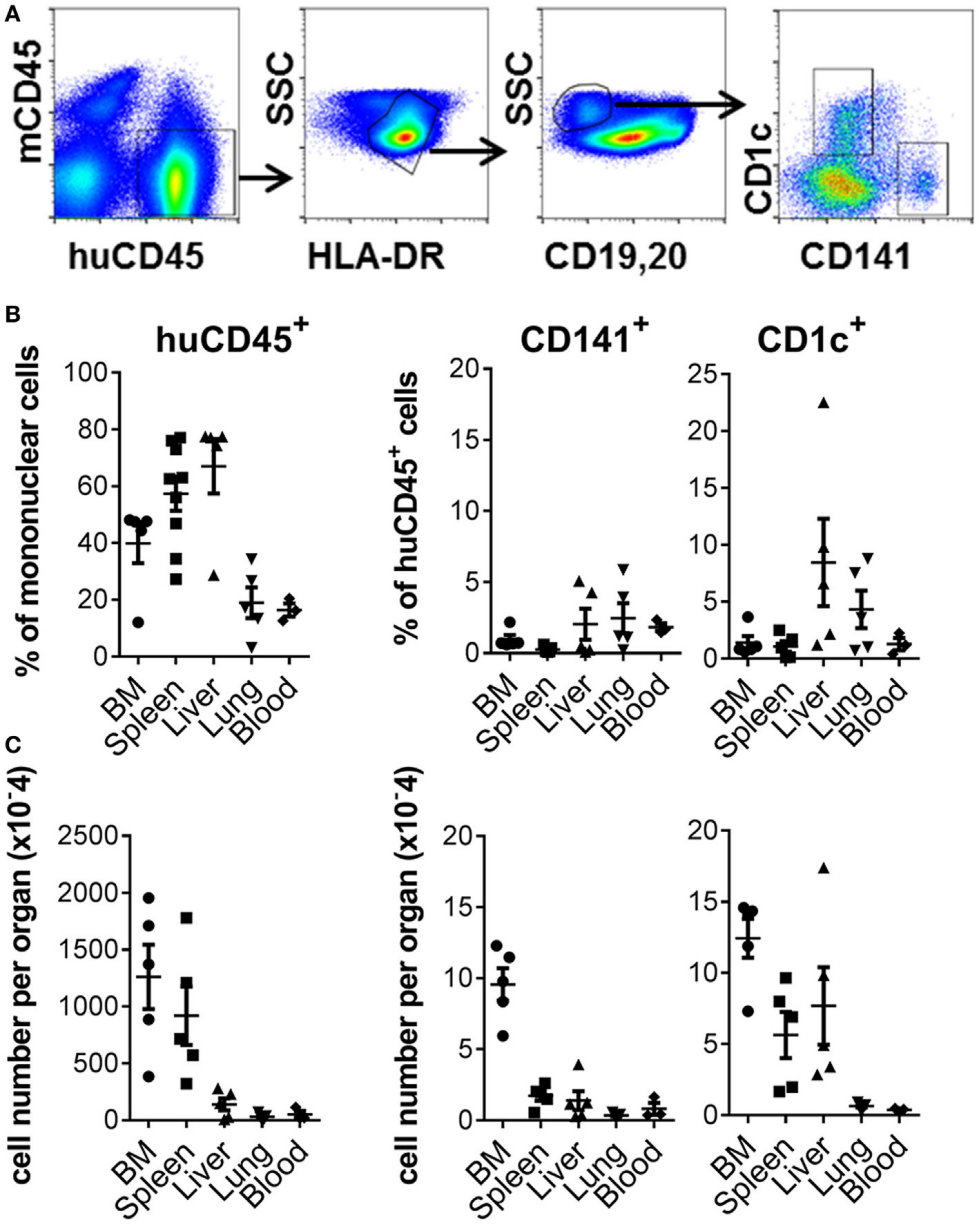
Development of CD141^+^ and CD1c^+^ dendritic cell (DC) in hu mice. **(A)** Gating strategy used to define DC subsets in the bone marrow (BM) of hu mice. After gating on live singlet cells, DCs were defined as hu CD45^+^ HLA-DR^+^, CD19/20^−^, and CD1c^+^ or CD141^+.^ **(B)** Frequency of human leukocytes, CD141^+^ DC and CD1c^+^ DC engrafted in the organs of NSG-A2 mice expressed as % mononuclear cells (huCD45) or as % hu CD45^+^ cells (CD141^+^ and CD1c^+^ DC). **(C)** Frequency of human leukocytes, CD141^+^ DC and CD1c^+^ DC expressed as absolute cell numbers per organ. BM data are quantitated per tibia and femur, and blood per ml.

Robust engraftment of human CD45^+^ cells was observed 10–14 weeks after reconstitution of 2–5-day-old NSG-A2 pups with human cord blood-derived CD34^+^ HSC (Figure 1A; Figure S1 in Supplementary Material). Human CD45^+^ cells were most prevalent in the spleen and liver, where they comprised 57.34% + 17.97 (mean + SD, *n* = 9) and 67% + 21.45 (mean + SD, *n* = 5) of mononuclear cells, respectively. Human CD45^+^ cells comprised 39.88% + 15.66 (mean + SD, *n* = 5) of mononuclear cells in the BM and could also be detected in lower proportions in the lungs and blood (Figure 1B). Human CD141^+^ and CD1c^+^ DC subsets were found in all organs examined, with the largest numbers residing in the BM compartment (Figure 1C). Human B cells, CD14^+^ monocytes, plasmacytoid DC, and CD4^+^ and CD8^+^ T cells also developed in this model (Figure S1 in Supplementary Material). Thus, NSG-A2 mice support robust, multi-lineage development of human leukocytes, including CD141^+^ and CD1c^+^ DC.

**Figure 2:**
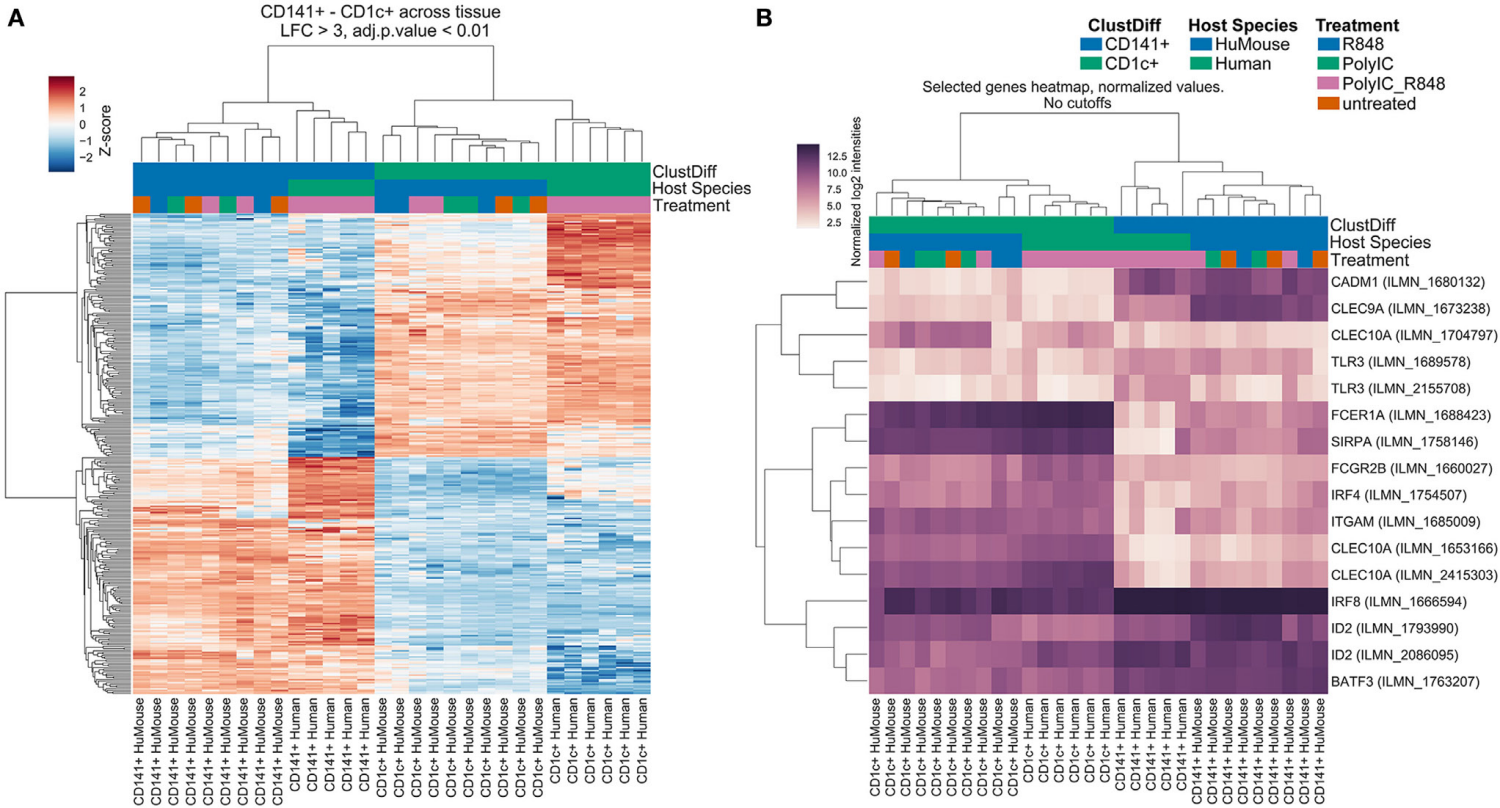
Hu mouse CD141^+^ dendritic cell (DC) and CD1c^+^ DC subsets cluster with their human blood counterparts. Hierarchical Cluster (Pearson correlation) of gene expression data showing **(A)** differentially expressed genes with a log-3 fold change (lfc) over one with adjusted *p* value < 0.01. **(B)** Expression of genes known to be associated with CD141^+^ DC and CD1c^+^ DC. Sample groups are colored at the bottom of the sample tree by ClustDiff (CD1c^+^ green; CD141^+^ blue); host species (humanized mouse green; human cells blue) and treatment (R848 alone blue; Poly I:C green; Poly I:C + R848 combination pink; untreated orange). The color of expression scores is scaled by Z-score, per row.

We compared transcriptomes of BM-derived CD141^+^ and CD1c^+^ DC from hu mice with equivalents from human blood (17). We identified 316 genes that were significantly differentially expressed (log-3 fold change, adjusted *p* < 0.01) between CD141^+^ and CD1c^+^ DC regardless of tissue source (Table S1 in Supplementary Material). Consistent with known DC functions, gene set enrichment analysis of these subsets ranked C-type lectins, endosomal TLR pathways, and the AIM2 inflammasome as most discriminating (Table S2 in Supplementary Material). Hierarchical clustering analysis of the differentially expressed genes revealed clustering of hu mouse CD141^+^ DC with human blood CD141^+^ DC, and hu mouse CD1c^+^ DC with human blood CD1c^+^ DC (Figure 2A). In an unsupervised clustering approach, the first component of a principal component analysis was concordant with batch, tissue, and species source (hu mice versus human blood). However, clustering of primary human CD141^+^ DC with hu mouse CD141^+^ DC, and CD1c^+^ DC across the two datasets with human peripheral blood monocytes was evident between the first and second principal components (Figure S3 in Supplementary Material).

Amongst the 156 genes preferentially expressed by hu mouse CD141^+^ DC and blood CD141^+^ DC were genes previously associated with this subset including *CLEC9A, CADM1, ID2, BATF3, RAB7B, AIM2, BTLA, SEPT3, CLNK*, and *GSAM* (Figure 2B; Table S1 in Supplementary Material). *IRF8, TLR3*, and *XCR1* were also more highly expressed by CD141^+^ DC (Figure 2B; Figure S4 in Supplementary Material). Genes known to be expressed by the CD1c^+^ DC lineage, including *IRF4, SIRPA, CLEC10A, FCGR2B, FCER1A, TLR5*, and *TLR7* were amongst the 160 top ranked differentially expressed genes in hu mouse and human blood CD1c^+^ DC (Figure 2B; Table S1 in Supplementary Material). CD1c^+^ blood DCs have recently been subdivided into two subsets defined as “non-inflammatory” or “CD1c A”, defined by expression of *FCER1A, CLEC10A*, and *FCGR2B*; and “inflammatory” or “CD1c B” defined by expression of *CD36* and *CD163* along with inflammatory genes including *CD14, S100A9, S100A8* (5). Hu mouse CD1c^+^ DC expressed much higher levels of the CD1c A non-inflammatory markers than hu mouse CD141^+^ DC, but also expressed the inflammatory markers *CD163, CD14, S100A9*, and *S100A8*, which were further upregulated after activation (Figure S5 in Supplementary Material). These data indicate that the hu mouse CD1c^+^ DC isolated here likely contain both inflammatory and non-inflammatory CD1c^+^ DC subsets. Because CD1c^+^ DC have known overlapping features with monocytes (20), we used differential expression analysis to identify genes that CD1c^+^ DC and monocytes co-express. More than 820 differentially expressed genes were identified by comparing hu mouse CD1c^+^ DC, blood CD1c^+^ DC, and blood monocyte subsets with CD141^+^ DC and pDC (log-3 fold change, adjusted *p* < 0.01, and Figure S6 in Supplementary Material). Genes shared between monocytes and CD1c^+^ DC included *CSF1R, SIRPA, IL1B, NLRP3, CASP1, FCGR2A*, and *FCGR2B*.

### R848 and Poly I:C Induce Common Gene Expression Profiles in CD141^+^ DC and CD1c^+^ DC *In Vivo*

**Figure 3:**
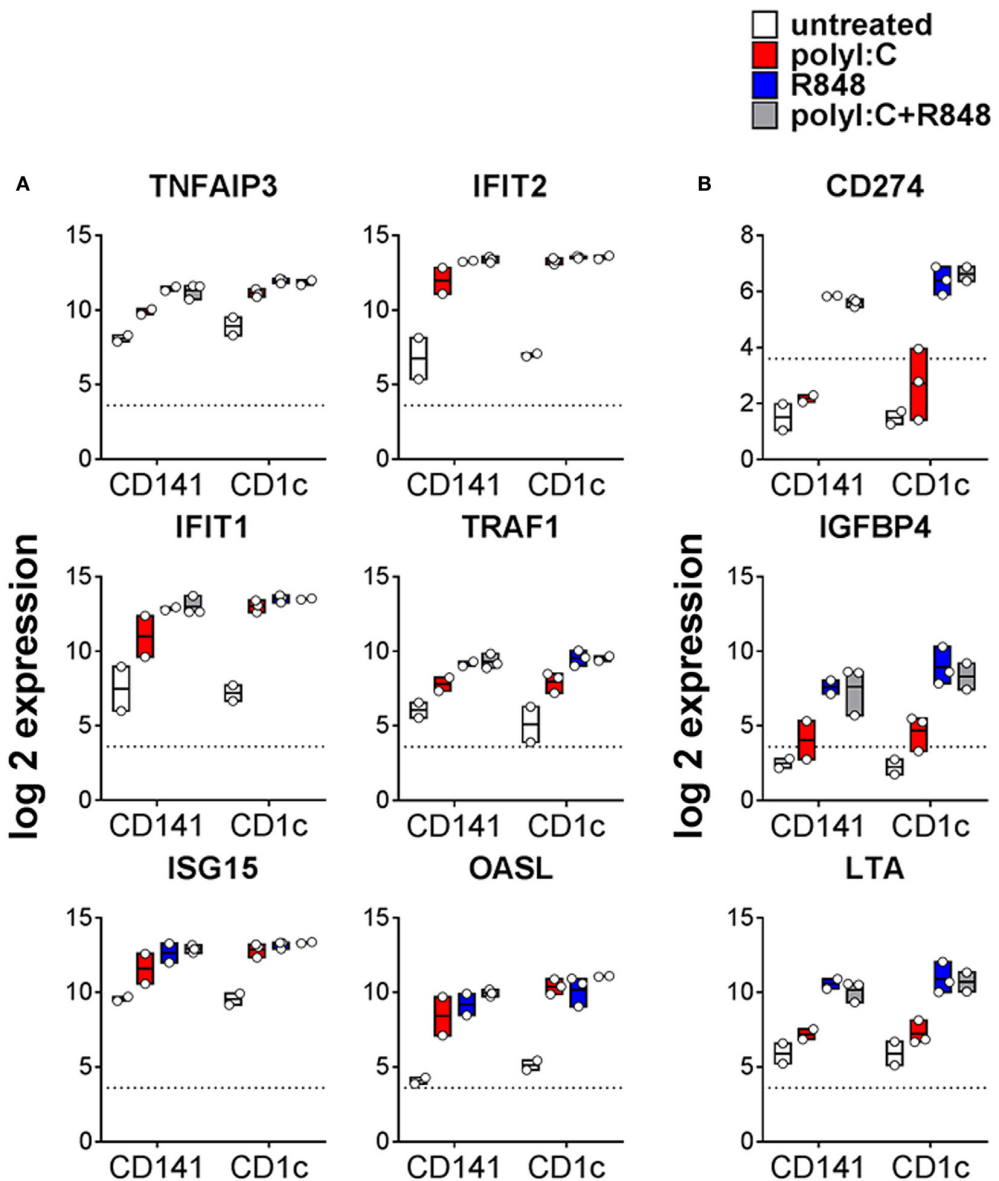
Changes in gene expression by hu mouse dendritic cells (DC) as a result stimulation with poly I:C and or R848 *in vivo*. Expression of CD1c^+^ DC and CD141^+^ DC genes following 2 h activation with poly I:C and/or R848 *in vivo* in hu mice. Boxes represent 25th–75th percentiles ± minimum and maximum values with line at the median from DC sorted from 2 to 3 individual mice. Expression of selected differentially expressed genes **(A)** upregulated by all TLR stimuli and **(B)** more highly expressed by R848 activated DC. Y-axis (normalized, log (2) expression).

TLR expression and maturation by human DC are well characterized *in vitro* but their responses *in vivo* are not. The TLR3 ligand, poly I:C, and the TLR7/8 ligand, R848, have been used as adjuvants to activate human DC alone or in combination (21, 28, 33, 34). Because cytokines can be detected in mouse serum within 1 h of adjuvant injection (35), we chose a 2 h time point to investigate the early changes in gene expression associated with activation of BM derived CD141^+^ DC and CD1c^+^ DC *in vivo*. A one-way AVONA analysis (adjusted *p* < 0.05) revealed differential expression of 408 genes as a result of activation. These genes were involved in immune signaling pathways including Tnfr2 signaling, IFNα/β signaling, Tnfr1 signaling (29% of pathway genes represented, *p* = 9.05E-04), canonical NF-κB pathway (17% of pathway genes represented, *p* = 0.027), RIG-I/MDA5, and TLR signaling (Figures 3A,B; Table S3 in Supplementary Material). Twenty five of these genes belong to a core set of NK-κB or IFN-stimulated genes (ISG) associated with DC maturation in both mouse and human irrespective of stimuli (Table S4 in Supplementary Material) (36). Most of the genes differentially expressed after activation were upregulated to similar degree by CD141^+^ DC and CD1c^+^ DC in response to all stimulatory conditions. These included *TNFAIP3, TRAF1, IFIT1, ITIF2, ISG15*, and *OASL* (Figure 3A). A small subset of genes were significantly upregulated by both CD141^+^ DC and CD1c^+^ DC in response to R848 alone or combined with poly I:C but were only marginally upregulated by poly I:C alone. These included immunoregulatory molecules *LTA, NFIL3, CD274*, and *IGFB4* (Figure 3B). Conversely, no genes were found to be significantly upregulated on both subsets activated with poly I:C that were not also upregulated by R848.

**Figure 4:**
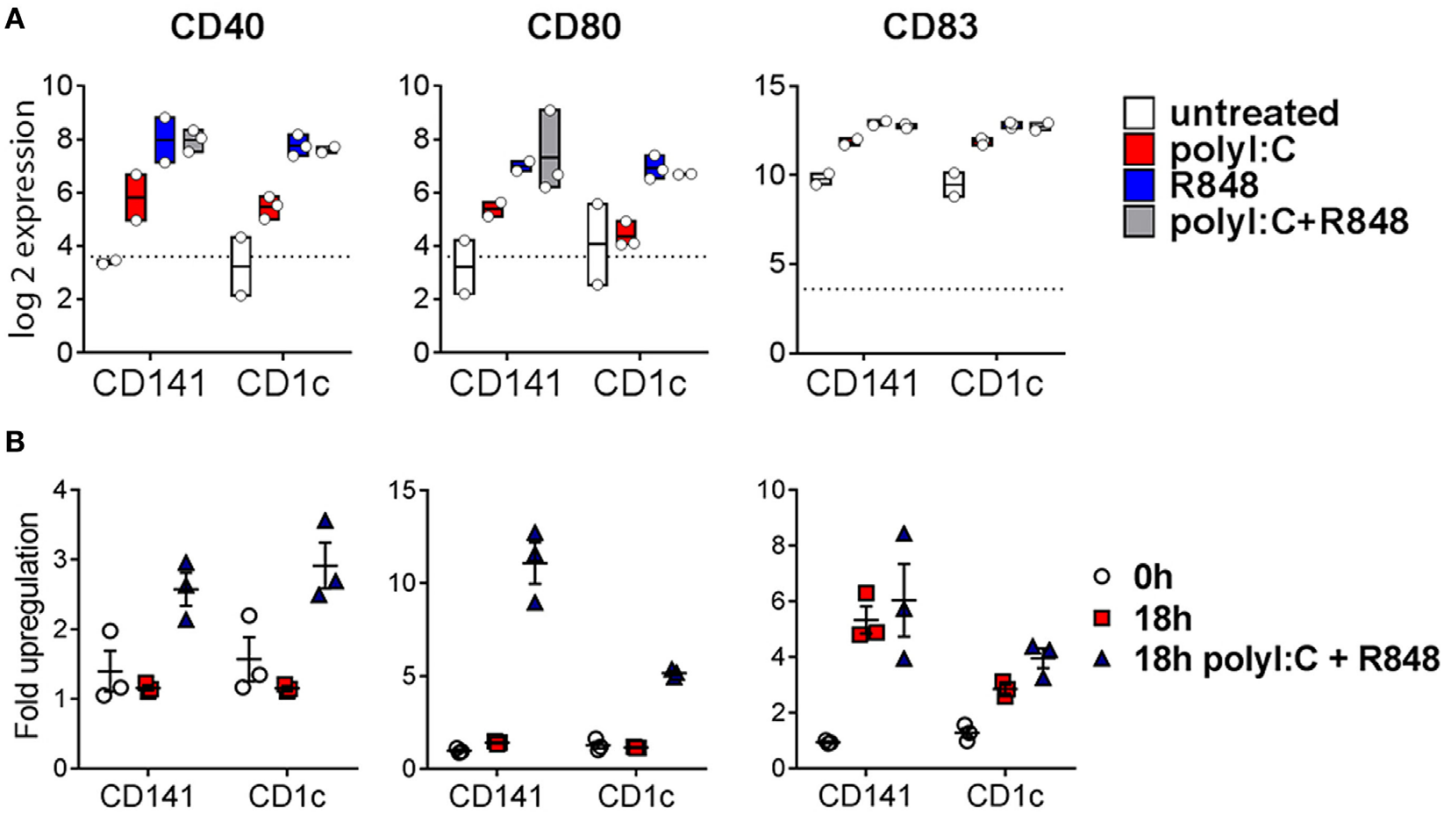
Expression of costimulatory molecules after activation of hu mouse CD141^+^ dendritic cells (DC) and CD1c^+^ DC. **(A)** Normalized log 2 expression of CD1c^+^ DC and CD141^+^ DC genes following 2 h activation with poly I:C and/or R848 *in vivo* in hu mice. Boxes represent 25th–75th percentiles ± minimum and maximum values with line at the median from DC sorted from 2 to 3 individual mice. **(B)** Cell surface protein expression of costimulatory molecules by purified DC subsets (0 h) and after 18 h culture *in vitro* in medium alone or with poly I:C + R848. Shown is the mean ± SEM fold upregulation of the mean fluorescence intensity of each costimulatory molecule relative to an isotype control.

**Figure 5:**
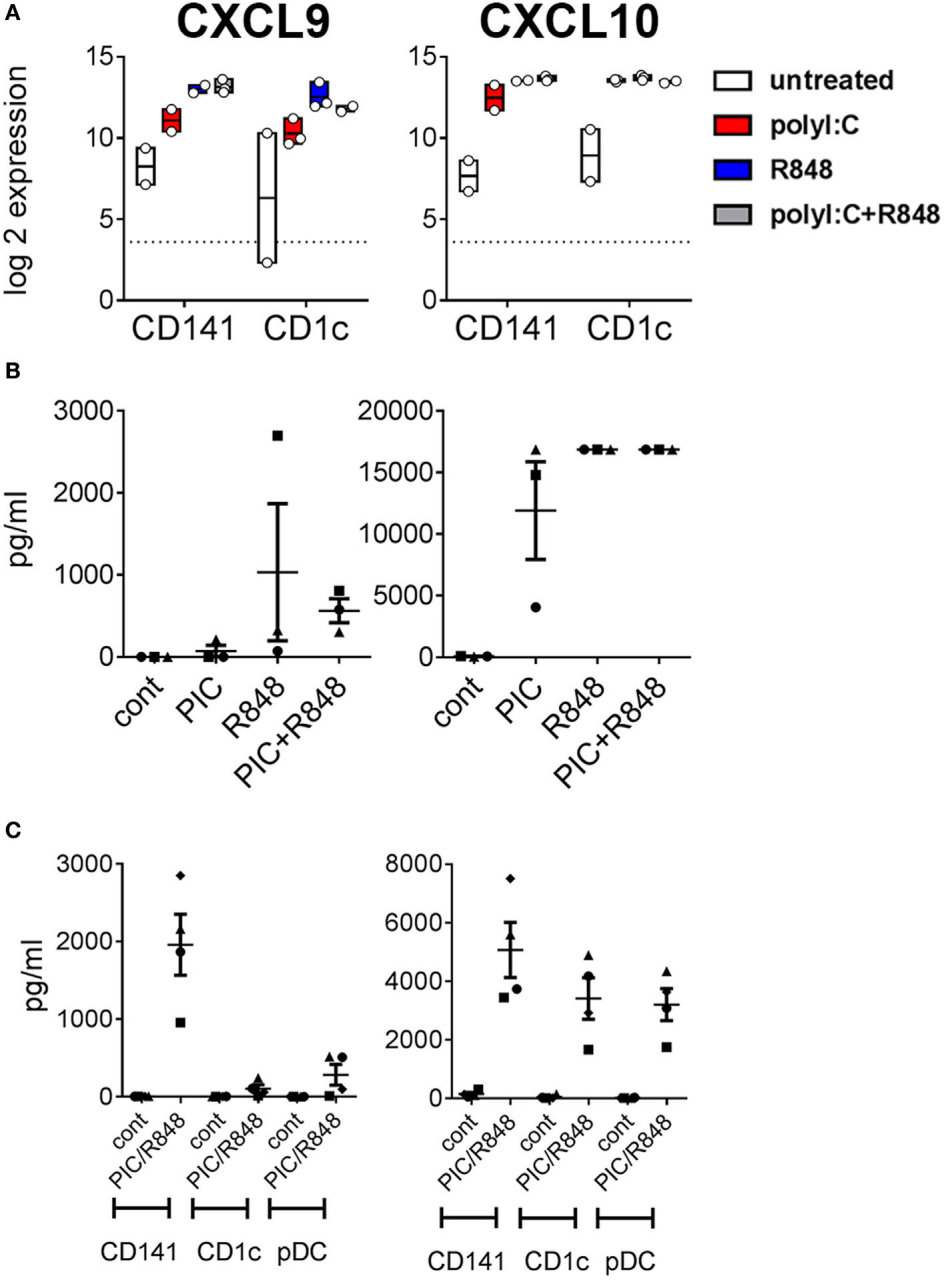
Expression of chemokines CXCL9 and CXCL10 by hu mouse CD141^+^ dendritic cells (DC) and CD1c^+^ DC. **(A)** Normalized log 2 expression of CD1c^+^ DC and CD141^+^ DC genes following 2 h activation with poly I:C and/or R848 *in vivo* in hu mice. Boxes represent 25th–75th percentiles ± minimum and maximum values with line at the median from DC sorted from 2 to 3 individual mice **(B)** Chemokine protein levels in the serum of hu mice 2 h after activation. **(C)** Production of chemokine protein by purified hu mouse DC 18 h after activation *in vitro*, versus control [cultured in medium alone *in vitro* (cont)].

Poly I:C and R848 alone or combined induced expression of costimulatory molecule genes *CD40, CD80*, and *CD83* to similar levels by both CD141^+^ DC and CD1c^+^ DC (Figure 4A). Gene expression correlated with increased protein expression of CD40, CD80, and CD83 on the surface of purified CD141^+^ DC and CD1c^+^ DC after *in vitro* activation (Figure 4B). However, cell surface expression of CD80 was significantly higher on activated CD141^+^ DC compared to activated CD1c^+^ DC, and while CD83 expression was also higher on activated CD141^+^ DC, this did not reach statistical significance. Similarly, chemokine genes *CXCL9* and *CXCL10* were upregulated by both DC subsets following activation but only *CXCL10* reached statistical significance (Figure 5A). Upregulation of *CXCL9* and *CXCL10* correlated with detectable levels of these chemokines in the serum of hu mice 2 h after activation (Figure 5B). *In vitro*, purified CD141^+^ DC were the main producers of CXCL9 while both subsets produced high levels of CXCL10 (Figure 5C). Collectively, these data highlight both common and distinct pathways of CD141^+^ DC and CD1c^+^ DC that are activated by R848, poly I:C, or the combination.

### CD1c^+^ DC Upregulate Th17 Promoting Cytokines after Activation *In Vivo* with R848

**Figure 6:**
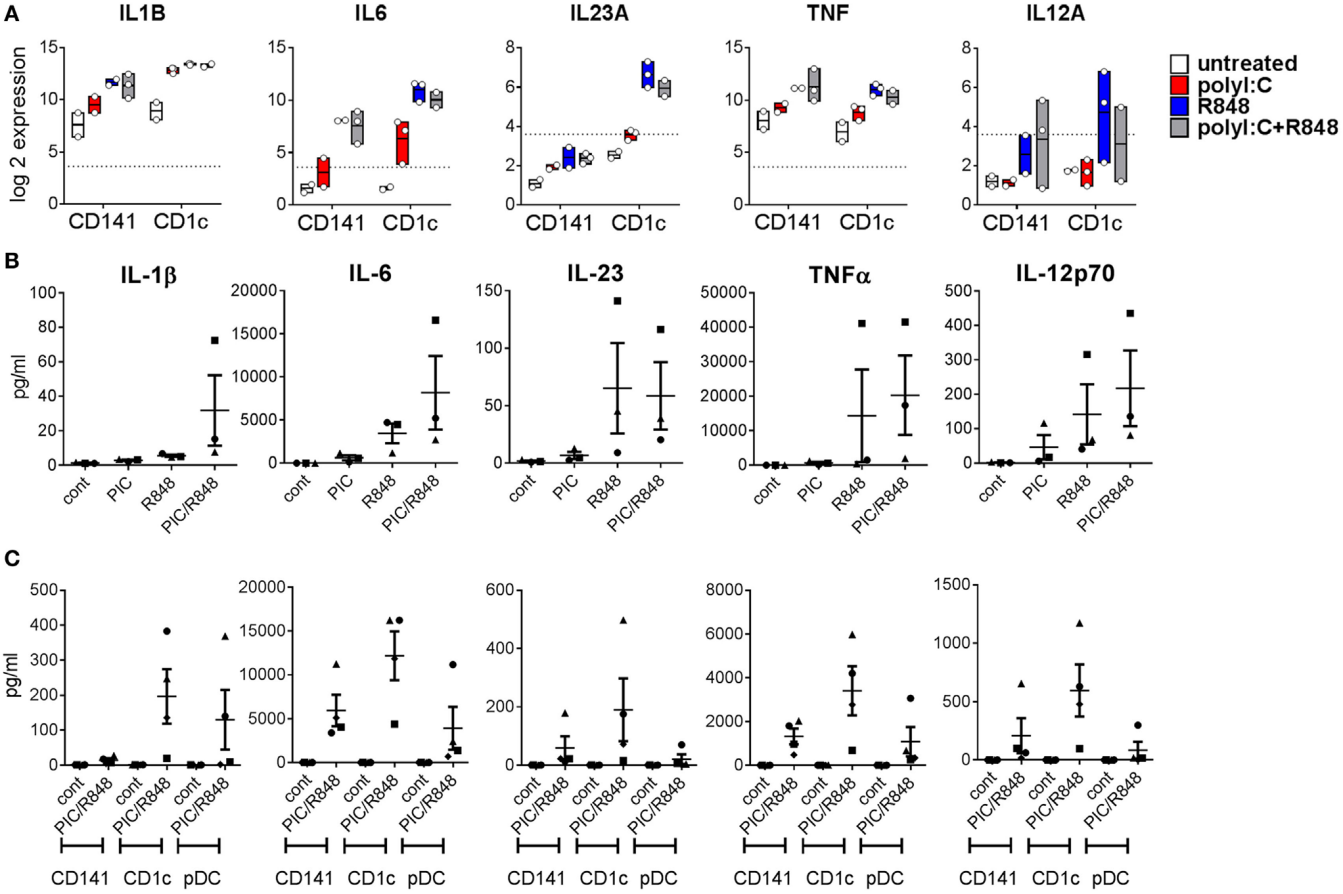
Expression of inflammatory cytokines by hu mouse CD141^+^ dendritic cells (DC) and CD1c^+^ DC. **(A)** Gene expression levels 2 h after activation of DC subsets *in vivo*. Data are presented as 25th–75th percentiles ± minimum and maximum values with line at the median of normalized log 2 gene expression by DC subsets sorted from 2 to 3 individual mice. **(B)** Cytokine levels in the serum of hu mice 2 h after activation. **(C)** Production of cytokines by purified hu mouse DC 18 h after activation *in vitro*, versus control [cultured in medium alone *in vitro* (cont)].

The mouse CD11b^+^ equivalents of CD1c^+^ DC promote Th17 responses (20). Consistent with this, we found genes encoding the Th17-promoting cytokines *IL1B* and *IL6* were upregulated by both BM derived DC subsets after activation, but levels expressed by activated CD1c^+^ DC were fourfold and eightfold higher than similarly activated CD141^+^ DC, respectively (Figure 6A). Importantly, expression of *IL23A* was 19-fold higher in CD1c^+^ DC activated with R848 alone or combined with poly I:C compared to similarly activated CD141^+^ DC. IL-1β, IL-6, and IL-23 were detectable in the serum of hu mice and although purified hu mouse CD1c^+^ DC produced higher amounts of these cytokines *in vitro* compared to CD141^+^ DC, the levels did not reach statistical significance (Figures 6B,C). TNF and IL-12A were similarly upregulated by *in vivo* activated CD141^+^ DC and CD1c^+^ DC with cytokines detectable in the serum. Higher levels of TNF and IL-12p70 were produced by *in vitro* cultured CD1c^+^ DC compared with CD141^+^ DC, although this did not reach statistical significance (Figure 6C).

### CD141^+^ DC Upregulate Type III IFN after Activation *In Vivo* with Poly I:C

**Figure 7:**
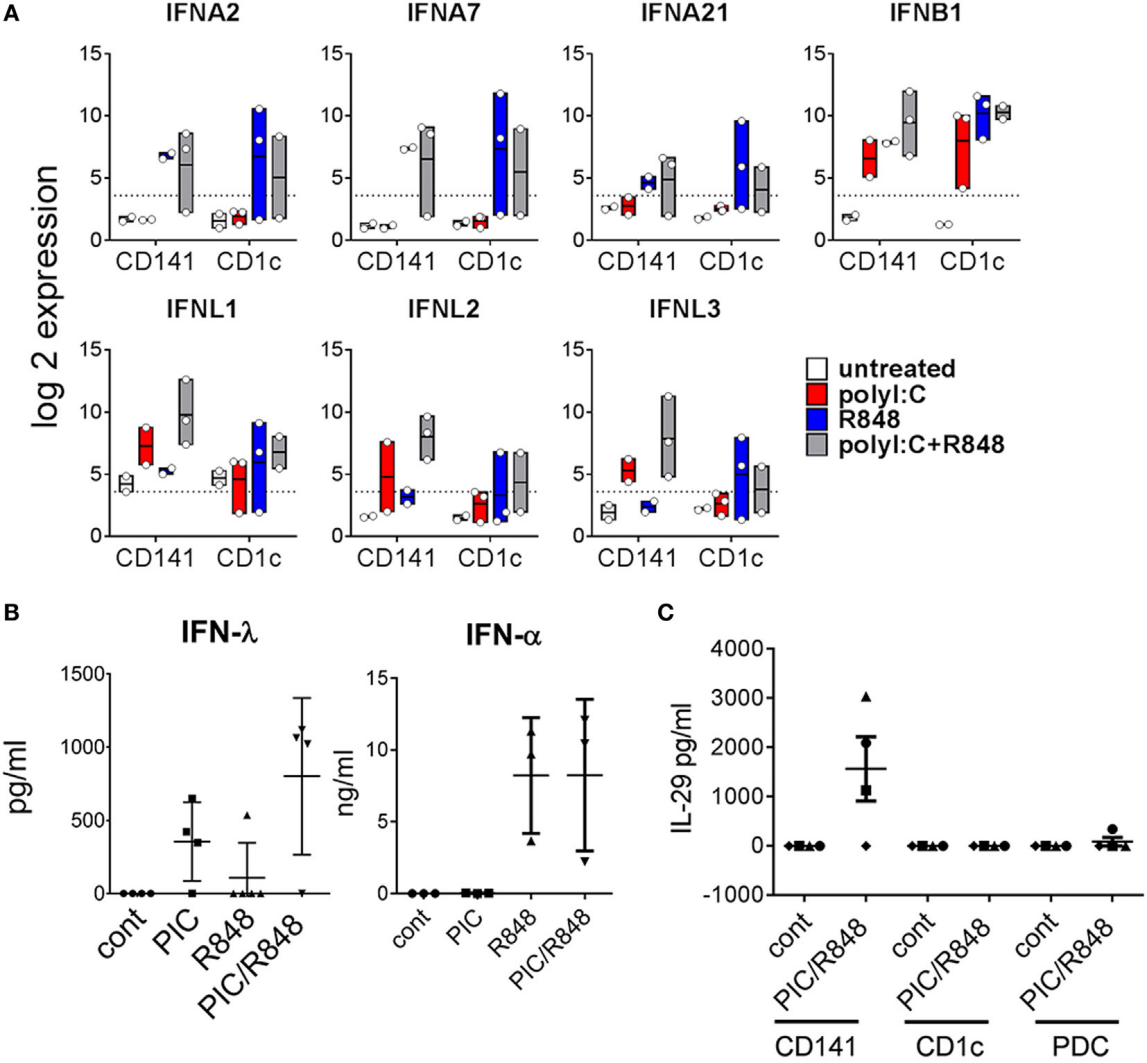
Expression of type I and type III IFN by hu mouse CD141^+^ dendritic cells (DC) and CD1c^+^ DC. **(A)** Expression of selected IFN genes by hu mice DC 2 h after activation *in vivo*. Data are represented as min–max values (line at mean) from 2 to 3 replicates for each condition. **(B)** Human IFNα and IFNλ in the serum of hu mice 2 h after activation. **(C)** IFNλ production by DC subsets purified from hu mice after stimulation with poly I:C and R848 *in vitro*, versus control [cultured in medium alone *in vitro* (cont)].

CD141^+^ DC have previously been shown to produce IFNα after poly I:C activation *in vivo* (28). Here, we found genes for IFNα subtypes *IFNA2, IFNA7*, and *IFNA21* were upregulated to a similar degree by both subsets, most notably in response to R848 alone or combined with poly I:C, while genes encoding most other IFNα subtypes were below detectable levels in both BM-derived DC subsets (Figure 7A). Although large amounts of IFNα were detectable in the serum of mice treated with R848 and R848 + poly I:C, no IFNα was detected in the serum of mice treated with poly I:C alone (Figure 7B). Moreover, IFNα was not detectable in the supernatants of purified CD141^+^ or CD1c^+^ DC activated *in vitro* (data not shown). *IFNB1* was upregulated by both subsets in response to all stimuli. CD141^+^ DC are known to produce large amounts of IFNλ in response to poly I:C (14) and consistent with this, IFNλ genes *IL28A, IL28B*, and *IL29* were expressed 13-, 17-, and 8-fold higher by poly I:C + R848-activated CD141^+^ DC compared to similarly activated CD1c^+^ DC (Figure 7A). This correlated with detection of IFNλ in the serum and in the supernatants of purified CD141^+^ DC activated *in vitro* (Figure 7C). These data confirm that CD141^+^ DC are major producers of IFNλ *in vivo*.

### CD141^+^ DC Upregulate *MYCL* after Activation and Prime Naïve CD8^+^ T Cells

**Figure 8:**
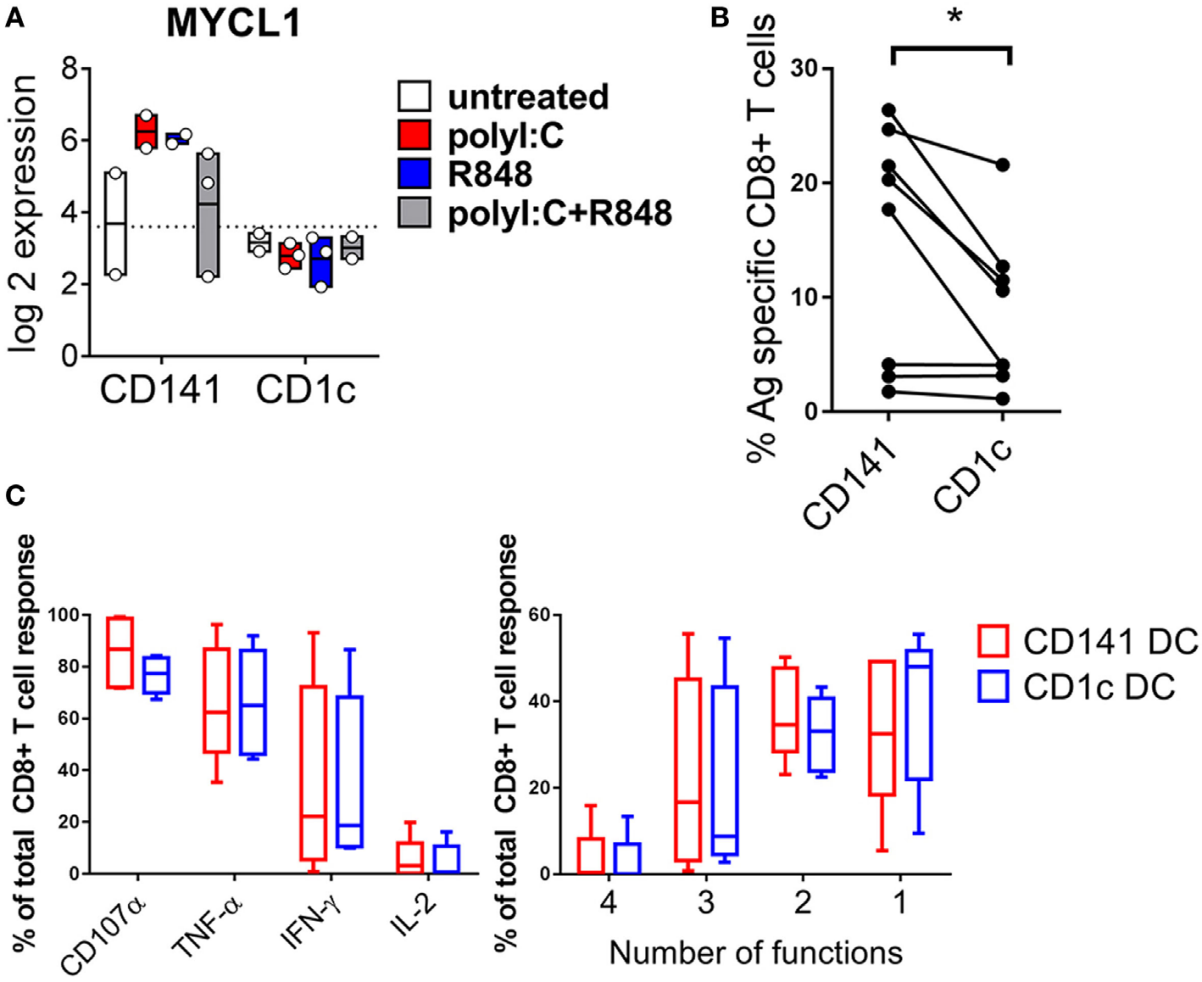
Increased expression of *MYCL* and CD8^+^ T cell priming capacity by activated CD141^+^ dendritic cells (DC). **(A)** Gene expression of *MYCL* 2 h after activation of DC subsets *in vivo*. **(B)** Priming of autologous naïve MART-1-specific CD8^+^ T cells by CD141^+^ DC and CD1c^+^ DC, shown as the percentage of CD8^+^ T cells staining for MART-1-specific pentamer. **(C)** Effector function of MART-1-specific CD8^+^ T cells primed by CD141^+^ DC and CD1c^+^ DC expressed as % of antigen (Ag)-specific cells producing effector molecules CD107a, TNF, IFNγ, or IL-2 (left) and % of Ag-specific CD8^+^ T cells simultaneously producing one or more effector molecules (right).

*MYCL* is required for optimal CD8^+^ T cell priming by mouse DC (37) and was expressed at 10-fold higher levels by CD141^+^ DC activated with either poly I:C or R848 compared to similarly activated CD1c^+^ DC (Figure 8A). This suggested an enhanced capacity for activated CD141^+^ DC to prime naïve CD8^+^ T cells that may be independent of their enhanced capacity for cross-presentation. To address the potential for enhanced priming ability by activated CD141^+^ DC, we purified human HLA-A2^+^ DC subsets from hu mice and pulsed them with the HLA-A2-restricted peptide from the melanoma Ag MART-1 after activation with combined poly I:C + R848. Peptide pulsed, activated CD141^+^ DC and CD1c^+^ DC were used to prime autologous, naïve cord blood CD8^+^ T cells. CD141^+^ DC primed a significantly higher percentage of MART-1-specific CD8^+^ T cells compared to CD1c^+^ DC (Figure 8B). However, there was no difference in the polyfunctional capacity of the T cells by either subset, as measured by production of effector molecules IFNγ, TNF, IL-2, and CD107a (Figure 8C).

## Discussion

In this study, we demonstrate that hu mice are a robust model to study human CD141^+^ DC and CD1c^+^ DC development and function *in vivo*. Using global transcriptome analysis, we showed that CD141^+^ DC and CD1c^+^ DC developing in the BM of hu mice closely resembled those of their human blood counterparts. Moreover, we have mapped the early genetic changes that human CD141^+^ DC and CD1c^+^ DC undergo following activation *in vivo*. Our data show that when activated with poly I:C or R848, alone or combined, both subsets undergo similar initial genetic programing events that include upregulation of a core set of genes broadly associated with DC maturation, in addition to upregulation of a more select number of subset-specific genes that confer functional specificity.

We used publically available datasets for human blood DC and monocyte subsets to establish the degree of similarity with human DC subsets developing in hu mouse. We identified a signature of 156 preferentially expressed genes that were shared by blood and hu mouse CD141^+^ DC regardless of their activation status, including many of the known hallmark genes such as *CLEC9A, CADM1, ID2*, and *BATF3* (11, 38, 39). Other hallmark genes including *IRF8, TLR3*, and *XCR1* were also confirmed to be expressed by hu mouse CD141^+^ DC but fell outside the stringent statistical threshold, either because the genes changed after activation or due to low probe hybridization (33, 40–42). These data demonstrate close genetic similarity between CD141^+^ DC developing in hu mice with those in human blood, providing further validation of hu mice as a powerful model to study human CD141^+^ DC biology. A recent study comparing the transcriptomes of human DC subsets in blood, spleen, thymus, tonsil, BM, and cord blood demonstrated that CD141^+^ DC in these organs are most strongly defined by ontogeny and are less influenced by their tissue environment (43). Cross presentation primarily by conventional CD11c^+^ DC in the BM has been demonstrated, which primed naïve CD4^+^ and CD8^+^ T cells and resulted in cytotoxic T cells; demonstrating also functional similarity (44, 45). Therefore, hu mice are likely to be a useful model that more generally represents the CD141^+^ DC lineage across the lymphohematopoietic system. FLT3L treatment was used to expand the number of human DC *in vivo* from humanized mice as per our previous published studies (30). Expansion of DC is performed by two injections of FLT3L 4 days apart prior to experimental treatments. This prior study demonstrated that the ratio of CD1c^+^ DC to CD141^+^ DC in humanized mice becomes more like that found in human blood (~8:1), and human spleens (~4:1) after FLT3L injections, and FLT3L-expanded DCs were functionally similar to their human DC counterparts. Thus, the abundance of CD141^+^ DC in hu mice, particularly after FLT3L administration, provides an excellent means of overcoming the lack of signatures obtained from published datasets (43). Indeed, we were able to recover sufficient cell numbers from the BM and RNA to obviate the need for amplification prior to hybridization. Although the transcriptional signatures of CD141^+^ DC in human skin and lung share similar ontogeny, they are also heavily influenced by tissue-derived signals (17, 43). CD141^+^ DC develop in the lung and liver of hu mice, but whether they are representative of those in non-lymphohematopoietic tissues remains to be determined.

CD1c^+^ DC in blood and hu mice shared preferential expression of 160 genes that included known hallmark genes associated with this subset such as *IRF4, SIRPA, CLEC10A, FCGR2B*, and *FCER1A*. However, consistent with other reports (17, 20), there were very few genes that were uniquely expressed by CD1c^+^ DC and many of the genes preferentially expressed by CD1c^+^ DC were shared with monocytes, suggesting overlapping functions between these two cell lineages. Although the degree of functional heterogeneity within the CD1c^+^ DC populations is unclear, single-cell RNA profiling has segregated blood CD1c^+^ DC into two further subtypes that are distinguished by expression of inflammatory genes (5). Genes discriminating both CD1c^+^ DC subsets were enriched in hu mouse CD1c^+^ DC and genes associated with the inflammatory CD1c^+^ DC subtype were further upregulated after activation in our dataset, suggesting that both subsets develop in hu mice. Thus, we conclude that the CD1c^+^ DC developing in hu mice are closely related to those in human blood and like CD141^+^ DC, may also be representative of this subset across the lymphohematopoietic system (43). However, as CD1c^+^ DC in non-lymphohematopoietic tissues appear to be even more influenced by their microenvironment than other DC subsets, the CD1c^+^ DC developing in hu mouse BM may not fully reflect the properties of these cells in other tissues.

Our study provides the first insights into the early genetic programing events that occur following activation of CD141^+^ DC and CD1c^+^ DC *in vivo*. We demonstrated a concordant gene expression pattern that occurred in both subsets in response to all stimuli. These genes were largely associated with ISG and NFκb signaling and many were found to belong to a core set of genes that are commonly induced following activation of mouse DC subsets and human DC cultured *in vitro* (34, 36). Our data extend these observations to demonstrate that upregulation of this core set of genes associated with DC maturation is also conserved in *in vivo* activated human CD141^+^ DC and CD1c^+^ DC, further validating the functional integrity of hu mouse DC. With the exception of a select panel of immunoregulatory genes that were more highly expressed following activation with R848, most genes upregulated on both DC subsets were induced to a similar degree by either poly I:C or R848 activation. Moreover, the combination of R848 and poly I:C did not further augment gene expression. Similar observations have been made on monocyte-derived DC, where the small proportion of genes specifically upregulated by combinatorial stimuli are only evident at later time points of 8 h post activation (46).

Only a small number of genes were found to be differentially regulated by CD141^+^ DC and CD1c^+^ DC in response to activators. Notably, activated CD1c^+^ DC expressed higher levels of *IL1B, IL6*, and *IL23A* compared to similarly activated CD141^+^ DC. CD1c^+^ DC purified from hu mice and activated *in vitro* produced higher levels of these cytokines compared to CD141^+^ DC, although this did not reach statistical significance. These observations support a role for CD1c^+^ DC in promoting Th17 responses similar to what has been reported for their mouse counterparts and human lung resident CD1c^+^ DC (20). Our data extend this to CD1c^+^ DC in the lymphohematopoietic system, suggesting that this is a conserved specialist function of this lineage that occurs within 2 h of activation. CD141^+^ DC and not CD1c^+^ DC upregulated *MYCL* following activation with poly I:C or R848 *in vivo*. Since *MYCL* has been shown to be essential for optimal CD8^+^ T cell priming by mouse DC (37) this suggests a similar function for human CD141^+^ DC. The higher expression of CD80, CD83, and CXCL9 by CD141^+^ DC is also consistent with a role in CD8^+^ T cell priming. Although peptide pulsed CD141^+^ DC were able to more efficiently expand naïve autologous Ag-specific CD8^+^ T cells compared to CD1c^+^ DC *in vitro*, the overall quality of the T cells in terms of cytokine polyfunctionality was similar and the role of individual human DC subsets in the induction of effector CD8^+^ T cell responses requires further clarification.

CD141^+^ DC were previously identified as being major producers of IFNα in response to poly I:C (28), however, our data did not provide evidence to support this *in vivo*. Although transcripts for a few IFN subtypes were found to be upregulated by both DC subsets and high levels of IFNα were detectable in the serum of hu mice after activation with R848, only *IFNB1* was found to be induced by poly I:C. In mice, most of the IFNα produced in response to poly I:C arises from non-hematopoietic cells (35), which is consistent with our hu mice, where the only human cells present are of hematopoietic origin. Type I IFN induction in DC occurs *via* an initial induction phase within 1 h followed by a feedback loop leading to a secondary amplification phase (47). Our data suggest that poly I:C induces IFNβ but not IFNα subtypes by CD141^+^ DC and CD1c^+^ DC, whereas R848 induces both IFNβ and IFNα during the first phase of induction. The production of IFNα by CD141^+^ DC observed by Meixlsperger et al. was measured at later time points, suggesting production during the secondary amplification phase. In contrast, increased expression of Type III IFN (IFNλ) genes, *IL28A, IL28B*, and *IL29* was evident in CD141^+^ DC activated with poly I:C alone or in combination with R848. This correlated with high levels of IFNλ secretion by this subset *in vitro* and is consistent with previous reports that CD141^+^ DC are major producers of IFNλ in response to poly I:C (14). Collectively our data support the use of hu mice as both a practical and valuable tool for characterizing human lymphohematopoietic CD141^+^ DC and CD1c^+^ DC function *in vivo*.

## Ethics Statement

Cord blood was obtained with written informed consent from the Queensland Cord Blood Bank with approval from the Mater Adult Hospital Human Ethics Committee. This study was carried out in accordance with the recommendations of the Australian code for the care and use of animals for scientific purposes (8th Edition). The protocol was approved by the University of Queensland Animal Ethics Committee.

## Author Contributions

YM, IR, and OH designed and performed the hu mouse experiments and analyzed the data. IV and CW analyzed and interpreted the bioinformatics data and wrote the manuscript. JM and YW designed, analyzed and interpreted data, KR conceptualized and designed the project, analyzed and interpreted data, and wrote the manuscript.

## Conflict of Interest Statement

The authors declare that the research was conducted in the absence of any commercial or financial relationships that could be construed as a potential conflict of interest.

## Supplementary Material

Table S1 List of genes differentially expressed by CD141^+^ dendritic cell (DC) and CD1c^+^ DC in hu mice and human blood.

Table S2 Reactome enrichment: list of pathways significantly enriched in hu mouse and human blood dendritic cell.

Table S3 Reactome enrichment analysis of differentially expressed genes in dendritic cell stimulated with poly IC, R848, and the combination *in vivo*.

Table S4 Differentially expressed genes in dendritic cell (DC) stimulated as a result of activation *in vivo*. Highlighted in yellow are genes previously identified as being upregulated in DC after activation.
