# Supplementary material for: Human CD141+ Dendritic Cell and CD1c+ Dendritic Cell Undergo Concordant Early Genetic Programming after Activation in Humanized Mice In Vivo
Source: Front Immunol. 2017 Oct 30;8:1419. doi: 10.3389/fimmu.2017.01419 (PMC5670352; doi:10.3389/fimmu.2017.01419)
Supplement: Supplementary file 1 [file presentation_1.pdf]

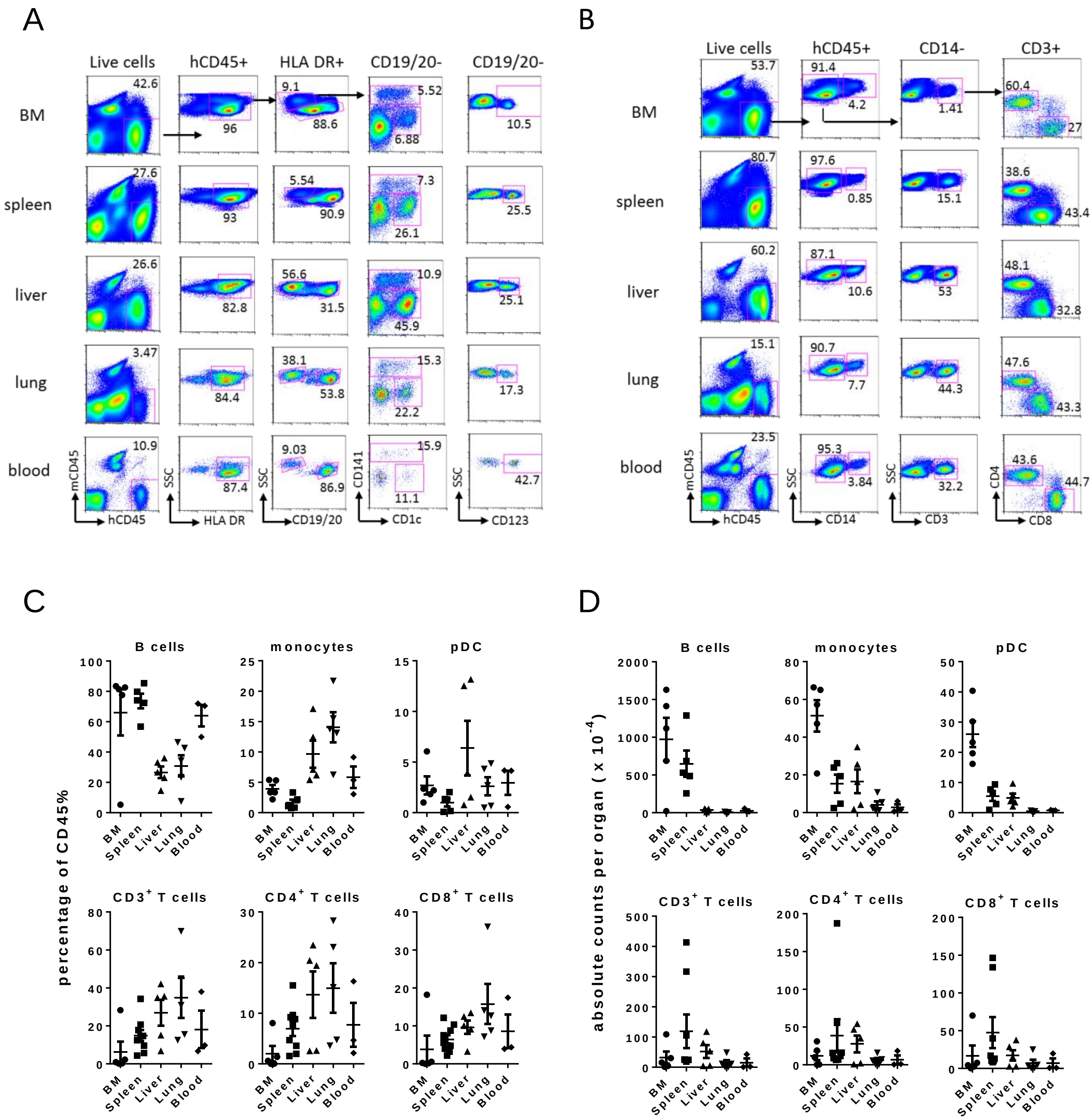

Figure S1. NSG-A2 mice support robust multi-lineage differentiation of human $CD34^+$ HSC in lymphoid and nonlymphoid organs. **A, B.** Live singlet cells were first gated on forward scatter and side scatter parameters followed by exclusion of live/dead$^+$ events (not shown). **A**. Gating strategy for B cells (huCD45$^+$ HLA-DR$^+$CD19/20$^+$), CD141$^+$ DC (huCD45$^+$ HLA-DR$^+$CD19/20$^-$ CD141$^+$), CD1c$^+$ DC (huCD45$^+$ HLA-DR$^+$CD19/20$^-$ CD1c$^+$), and plasmacytoid DC (huCD45$^+$ HLA-DR$^+$CD19/20$^-$ CD123$^+$). **B**. Gating strategy for monocytes (huCD45$^+$ CD14$^+$), CD4$^+$ T cells (huCD45$^+$ CD14$^-$ CD3$^+$ CD4$^+$) and CD8$^+$ T cells (huCD45$^+$ CD14$^-$ CD3$^+$ CD8$^+$). **C,D.** Frequency of leukocyte subsets expressed as **C.** absolute cell counts per organ (single tibia and femur for bone marrow (BM) and per ml for blood) and **D.** as % of huCD45$^+$ leukocytes. Each point represents and individual hu mouse.

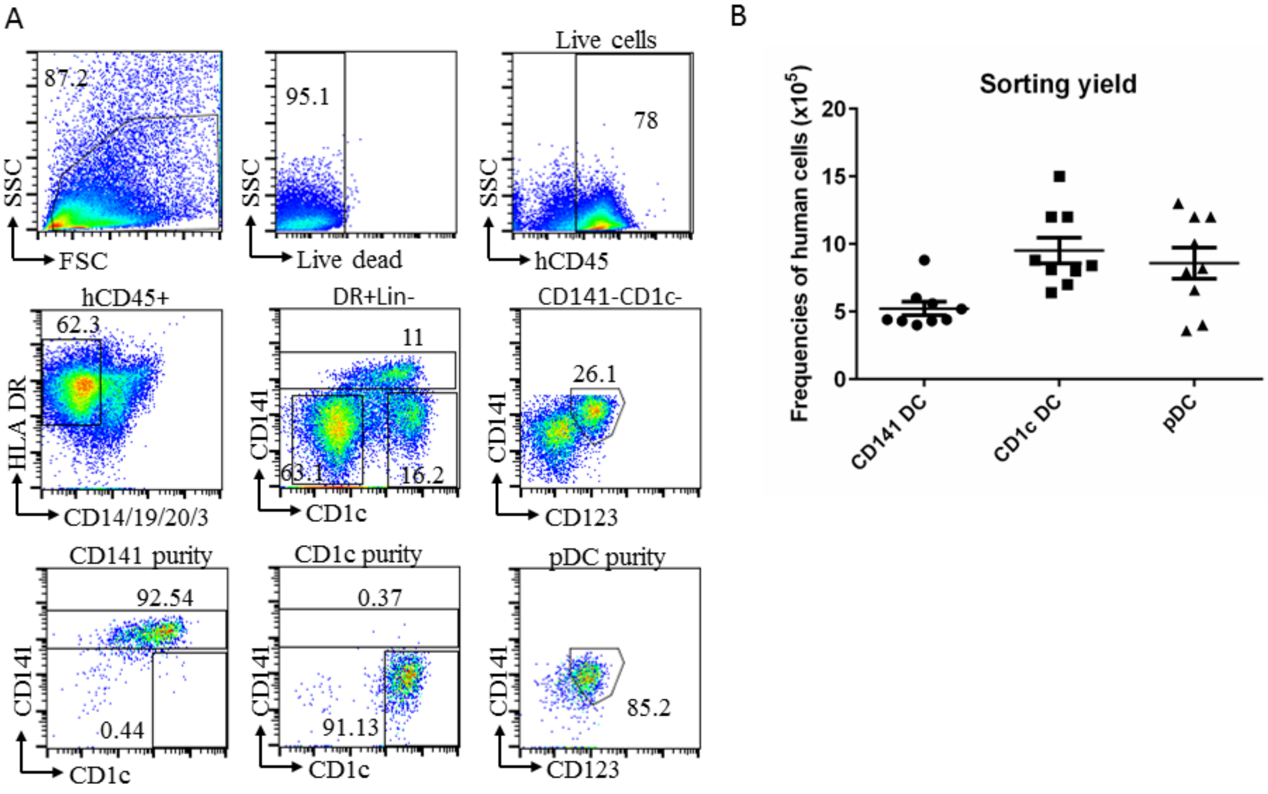

Figure S2: Sorting strategy for DC subsets and sorting yields. BM was isolated from FLT3-L injected humanised mice. DC subsets were sorted from lineage (mCD45, CD34, CD3, CD14, CD19, CD20) depleted BM. **A.** Dead cells were excluded by staining with Live Dead Aqua. DC were gated as hCD45+HLA DR+Lin- (CD3, CD14, CD19, and CD20) cells. DC subsets were sorted based on expression of CD141, CD1c and CD123. **B.** Sorting yield for each DC subsets.

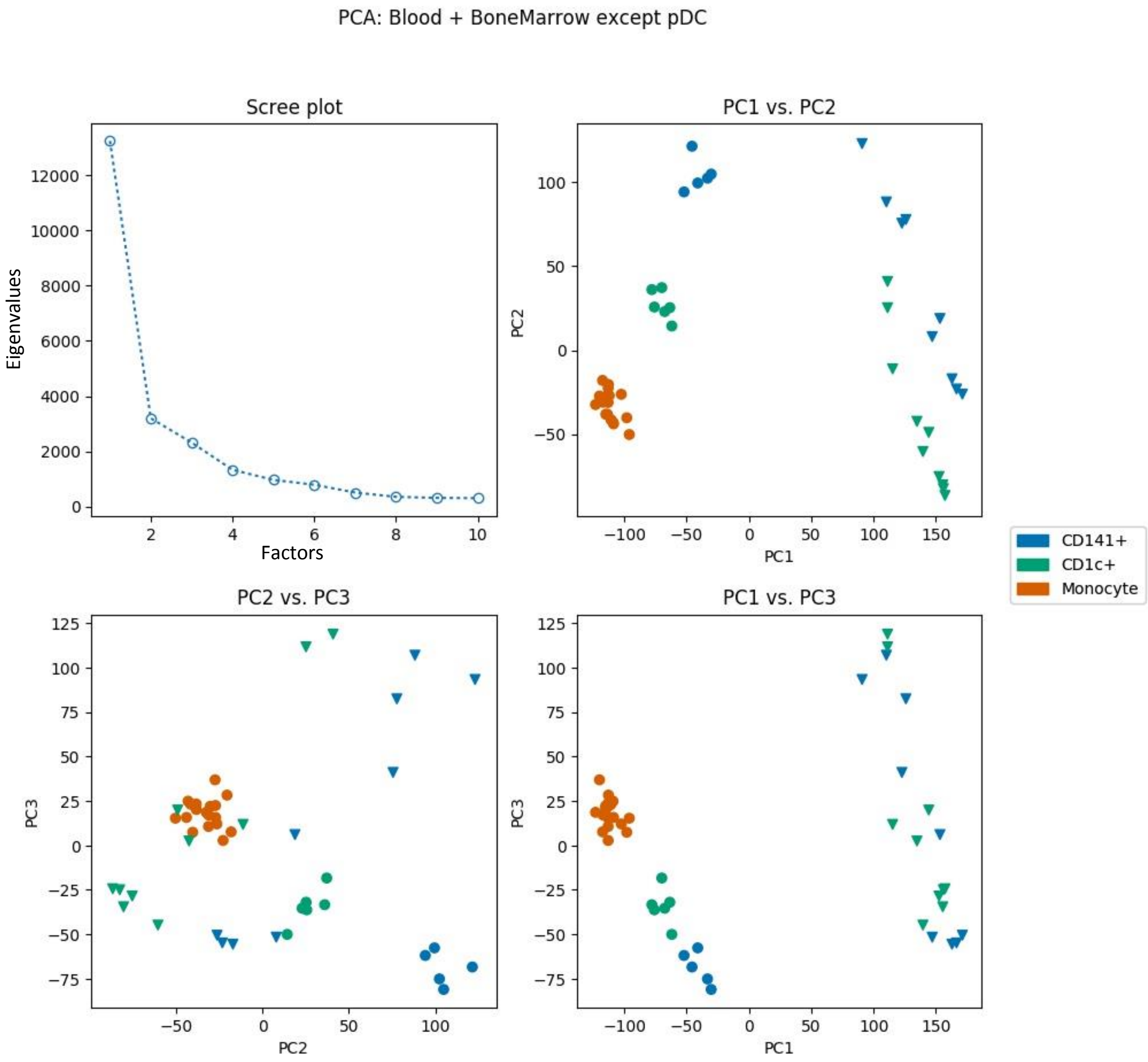

Figure S3: PCA analysis of CD1c v CD141 in hu mouse and blood. Triangles are hu mouse DC and circles are human blood DC and monocytes.

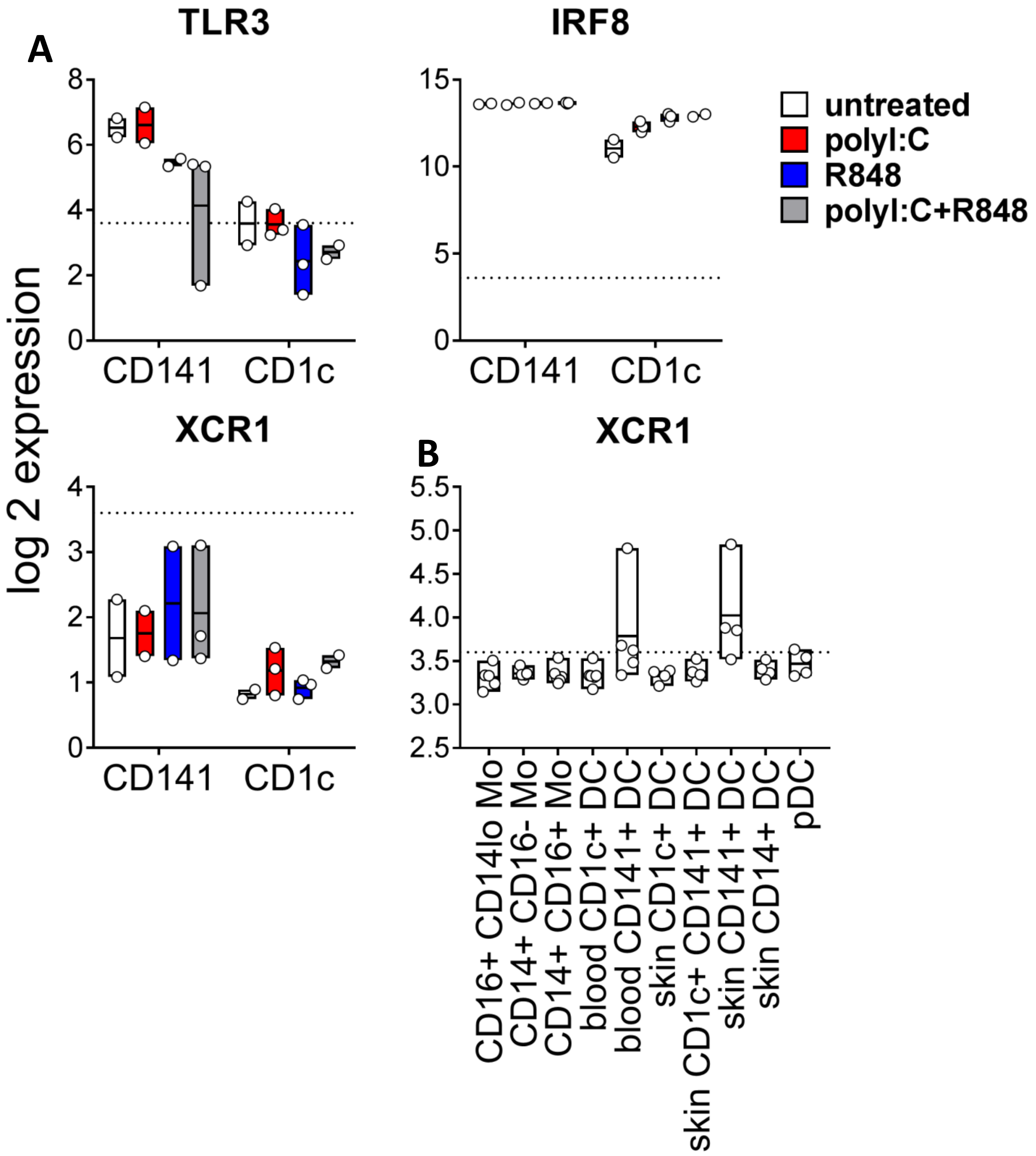

Figure S4: A. Expression of CD141⁺ DC associated genes TLR3, IRF8 and XCR1 that fell outside the strict log-3 fold change (adjusted $p < 0.01$) statistical threshold set for differential expression by humanised mouse DC. **B.** XCR1 expression by blood DC and monocyte subsets (data obtained from GSE35457 (Haniffa et al. 2012).

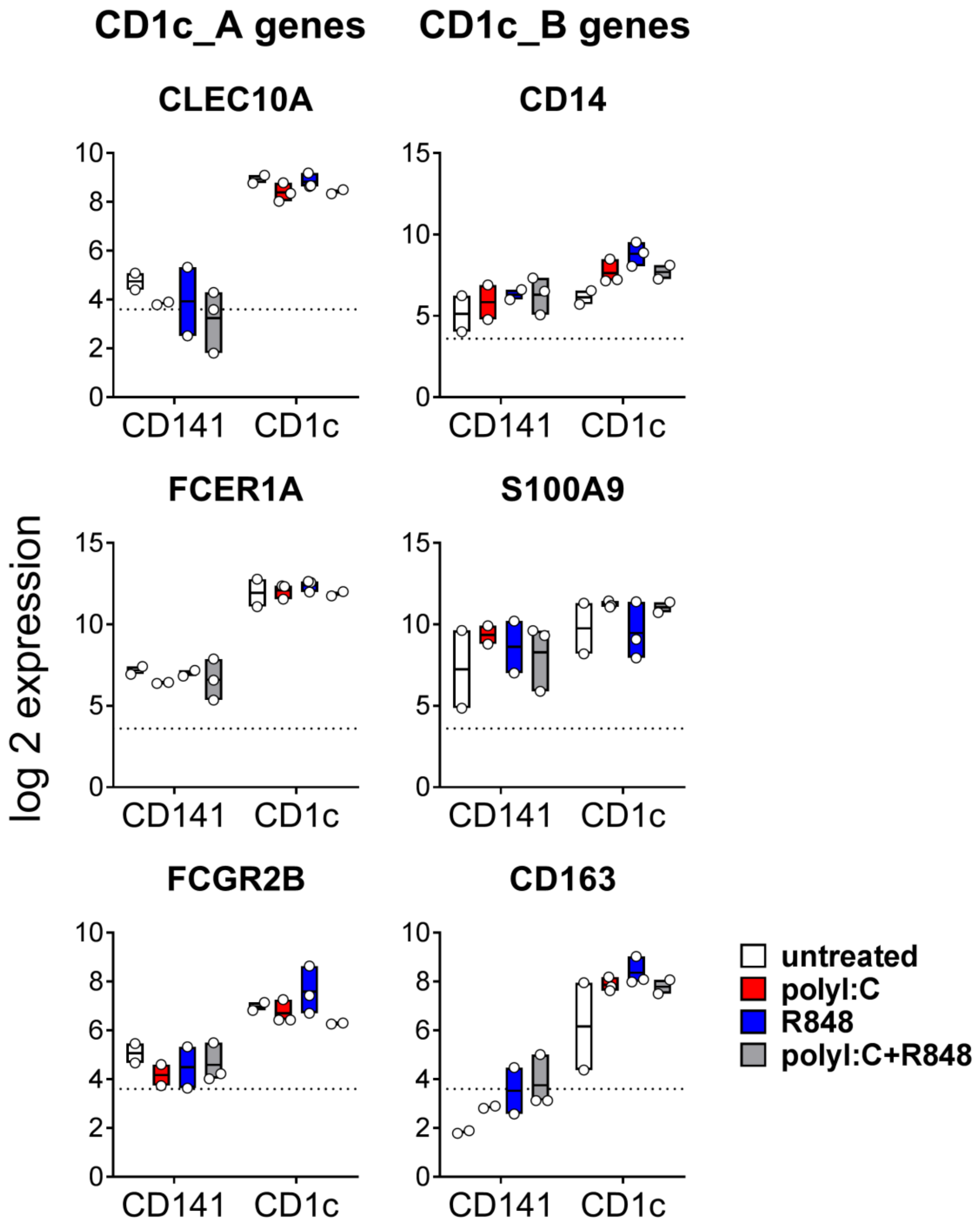

Figure S5: Expression of genes associated with distinct CD1c^{+} DC subsets, non-inflammatory CD1c_A and inflammatory CD1c_B.

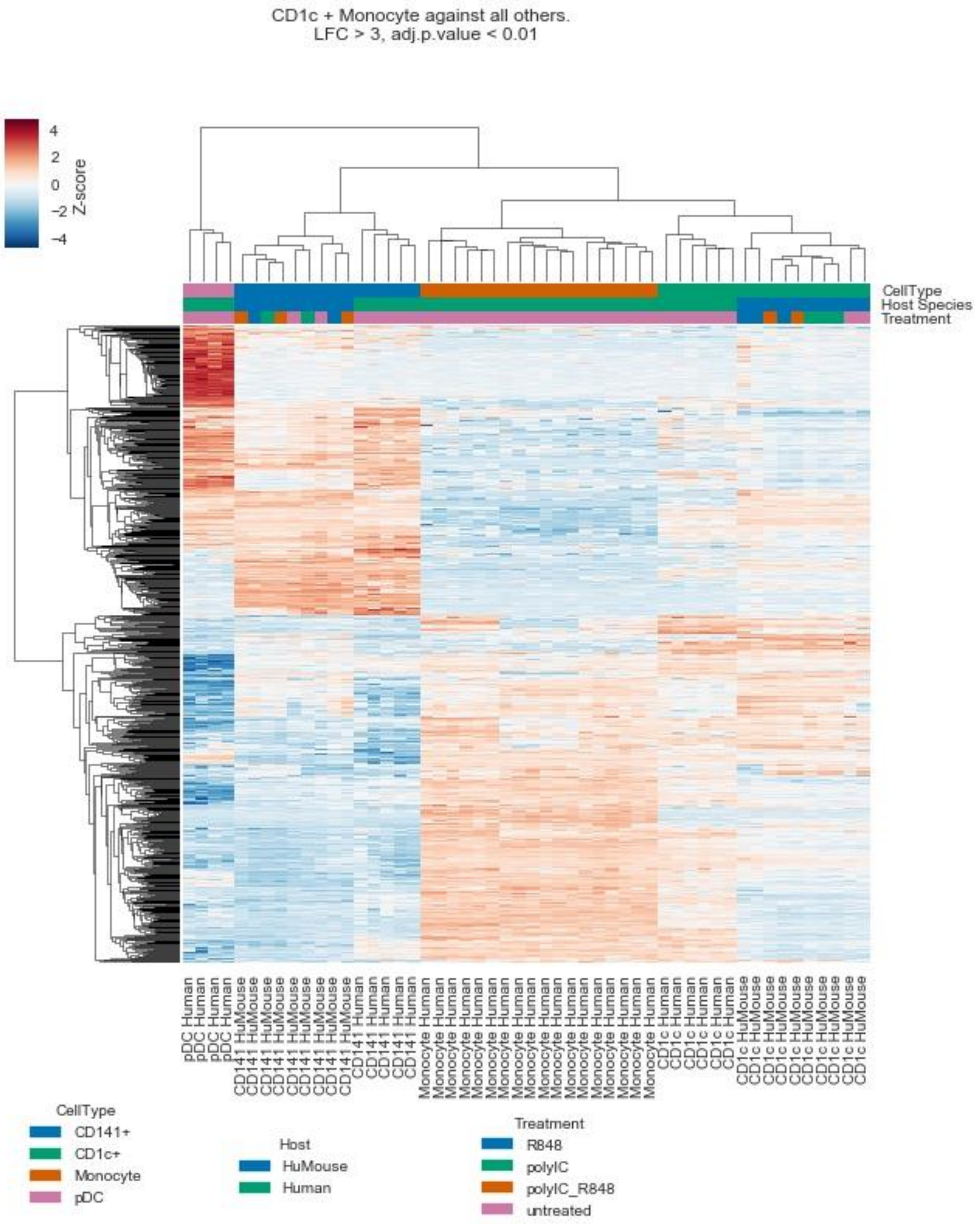

Figure S6: Heatmap showing genes differentially expressed by blood monocytes, CD1c^{+} DC and humanized mouse CD1c+ DC compared with other subsets (log-3 fold change, adjusted p.value < 0.01). Sample groups are colored at the bottom of the sample tree by ClustDiff (CD1c+ green; CD141+ blue); Host species (Humanised mouse green; human cells blue) and Treatment (R848 alone blue; Poly I:C green; Poly I:C + R848 combination pink; untreated orange). The color of expression scores is scaled by Z-score, per row.
